# Insomnia as an Independent Behavioral Correlate of Estimated Type 2 Diabetes Risk: Evidence from Three Validated Risk Assessment Scales

**DOI:** 10.5041/RMMJ.10579

**Published:** 2026-07-31

**Authors:** José Luis Ribes Valles, Pedro Juan Tárraga López, Ángel Arturo López González, Irene Coll Campayo, Carla Busquets-Cortés, José Ignacio Ramírez-Manent

**Affiliations:** 1 Clinical Analysis Laboratory, Son Llatzer Hospital, Palma, Spain; 2 Faculty of Medicine, University of Castilla-La Mancha, Albacete, Spain; 3 Faculty of Dentistry ADEMA University School, Palma, Spain; 4 Primary Care Balearic Islands Health Service, Palma, Spain; 5 Faculty of Medicine, University of the Balearic Islands, Palma, Spain

**Keywords:** Insomnia, lifestyle, socioeconomic factors, predictive value of tests, sleep initiation and maintenance disorders, type 2 diabetes mellitus

## Abstract

**Background:**

Sleep disturbances, particularly insomnia, are increasingly recognized as behavioral determinants of type 2 diabetes (T2DM). However, their contribution to validated diabetes risk scores beyond traditional sociodemographic and lifestyle factors remains insufficiently explored.

**Objective:**

To examine the association between insomnia severity, sociodemographic variables, lifestyle habits, and estimated T2DM risk using three validated non-invasive risk assessment scales.

**Methods:**

A cross-sectional study was conducted among 84,898 Spanish workers aged 18–69 years undergoing occupational health evaluations (2021–2024). Sociodemographic and behavioral data were collected through standardized questionnaires, including adherence to the Mediterranean diet (MEDAS-14), physical activity (IPAQ-SF), and smoking status. Insomnia was assessed using the Insomnia Severity Index (ISI). Diabetes risk was estimated using three validated non-invasive risk-assessment tools: the Finnish Diabetes Risk Score, the QDScore/QDiabetes, and the Trinidad Risk Assessment Questionnaire for Type 2 Diabetes Mellitus. As these instruments were developed and validated in different populations, comparisons of absolute risk categories across scales should be interpreted with caution. Multivariable logistic regression models adjusted for age, sex, social class, smoking, diet, and physical activity were used to estimate odds ratios (ORs) and 95% confidence intervals (CIs).

**Results:**

Severe insomnia was associated with approximately 2.1–2.6-fold higher odds of high estimated diabetes risk across all three scales in the pooled analyses, with significant trends across categories of insomnia severity (all *P* for trend <0.001). This association persisted across sex and activity strata. Adding ISI modestly improved model discrimination (ΔAUC ≈ +0.015; *P*≤0.003) and reclassification (continuous net reclassification improvement [NRI], 6.5%–8.1%).

**Conclusions:**

Insomnia severity was independently associated with higher estimated risk of T2DM beyond measured sociodemographic and lifestyle determinants. Incorporating sleep health—via brief tools such as the ISI—into diabetes risk assessment could enhance early prevention strategies in clinical and occupational settings.

## INTRODUCTION

Type 2 diabetes mellitus (T2DM) remains a major global health challenge, with sustained growth in prevalence across all regions and projections for substantial further increases over the coming decades.^1^ Beyond biological risk, a broad array of social determinants and behavioral factors shape T2DM risk and outcomes. Lower socioeconomic position—including lower educational attainment, income, and occupational status—has been consistently associated with higher T2DM incidence, highlighting the role of social gradients in diabetes epidemiology.^2^ Contemporary scientific reviews emphasize that social determinants of health (SDOH)—such as neighborhood deprivation, food and built environments, access to care, discrimination, and structural racism—operate across the life course to influence both the development and management of T2DM.^3^

Lifestyle factors remain central, and robust evidence links greater physical activity with lower T2DM risk across activity domains and intensities.^4^ Diet quality also shows consistent associations with T2DM incidence and prognosis, and multi-component “healthy lifestyle” profiles (e.g. non-smoking, healthy diet, adequate activity, and weight control) confer large relative risk reductions for incident T2DM and diabetes-related mortality.^5^–^7^ In parallel, cigarette smoking—still prevalent in many settings—raises T2DM risk by ~30%–40% and adversely affects insulin sensitivity and β-cell function, underscoring its continuing importance as a modifiable exposure in diabetes prevention strategies.^6^

Non-invasive risk scores synthesize sociodemographic and behavioral information to enable scalable identification of high-risk individuals in community and primary-care settings. The Finnish Diabetes Risk Score (FINDRISC), QResearch-derived Diabetes Risk Score (QDScore), and QResearch Diabetes Risk Prediction Algorithm (QDiabetes) exemplify widely used tools that integrate age, adiposity, family history, medication use, and lifestyle habits and, in the case of QDScore and QDiabetes, ethnicity and deprivation, to estimate 10-year T2DM risk without laboratory testing.^8^–^10^ Such scores facilitate targeted counseling, lifestyle intervention, and efficient deployment of confirmatory testing.

Sleep health has emerged as an additional—and often overlooked—behavioral domain relevant to T2DM prevention. Meta-analyses and large cohorts indicate U-shaped associations between habitual sleep duration and T2DM incidence, with the lowest risk near 7–8 hours and elevated risk among short and long sleepers; beyond duration, difficulties initiating or maintaining sleep (insomnia symptoms) also predict incident T2DM.^11^–^13^ Recent evidence using device-measured sleep and activity suggests that short sleep independently elevates T2DM risk, while sufficient physical activity may partially mitigate this excess risk.^11^,^12^ These findings complement broader literature connecting inadequate or disturbed sleep with insulin resistance, dysregulation of appetite and adiposity, and adverse cardiometabolic profiles.^11^–^15^

Taken together, contemporary diabetes prevention should consider the joint and potentially interactive effects of sociodemographic context, classic health behaviors (diet, activity, smoking), and sleep health. Evaluating how these domains collectively relate to established T2DM risk scores can clarify pathways of risk stratification, identify high-leverage targets for intervention, and inform pragmatic, non-invasive screening strategies adaptable to diverse populations.^3^–^10^,^12^–^13^ The present study therefore examines the association of sociodemographic variables, health habits (including smoking, physical activity, and diet), and insomnia-related sleep disturbances with validated non-invasive diabetes risk scales, aiming to refine population-level risk assessment and guide prevention efforts.^3^–^10^,^11^–^13^

## METHODS

### Study Design and Population

This cross-sectional study was conducted within the framework of a national occupational health surveillance program in Spain between January 2021 and December 2024. Data were collected during routine medical examinations carried out by occupational health professionals from various Spanish companies. Participants were employees from the industrial, commercial, and service sectors across several Spanish Autonomous Communities.

All data were anonymized before analysis in accordance with the General Data Protection Regulation (EU 2016/679) and Spain’s Organic Law 3/2018. The study protocol was approved by the Balearic Islands Research Ethics Committee (CEI-IB; protocol number IB 4383/20), and written informed consent was obtained from all participants.

### Eligibility Criteria

Inclusion criteria were: (1) age 18–69 years; (2) active employment at the time of assessment; and (3) complete data for sociodemographic, lifestyle, and sleep variables. Exclusion criteria were: (1) pregnancy; (2) known metabolic or endocrine disease (e.g. diabetes, thyroid disorders); (3) incomplete records; or (4) duplicated or inconsistent identifiers. After exclusions, 84,898 workers were included for analysis, consistent with prior investigations using this occupational database.^16^

### Sociodemographic Variables

Sociodemographic characteristics included age, sex, and occupational social class, determined according to the Spanish Society of Epidemiology classification based on the National Classification of Economic Activities (CNAE-11). Categories were: Class I (managers and professionals), Class II (intermediate occupations), and Class III (manual and routine workers).^17^

### Lifestyle Habits

Health behaviors were self-reported using validated questionnaires.

Dietary quality was assessed with the 14-item Mediterranean Diet Adherence Screener (MEDAS-14), validated for Spanish adults. Scores ≥9 indicated adequate adherence to the Mediterranean diet.^18^,^19^

Physical activity was evaluated with the International Physical Activity Questionnaire–Short Form (IPAQ-SF), classifying participants as “physically active” or “inactive” per standardized protocols.^20^

Smoking status was dichotomized as current smoker or non-smoker.^21^

### Sleep Assessment

Insomnia symptoms were measured using the Insomnia Severity Index (ISI), a seven-item validated tool for assessing subjective sleep difficulties and their impact on daytime functioning. Scores range from 0 to 28 and were categorized as: no insomnia (0–7), subthreshold (8–14), moderate (15–21), and severe (22–28).^22^,^23^

The ISI has demonstrated high internal consistency (Cronbach’s α >0.85), test–retest reliability, and validity in occupational and population studies.^24^ Sleep health was analyzed both categorically and as a continuous ISI score to explore dose–response associations.

### Diabetes Risk Assessment

Risk of type 2 diabetes (T2DM) was estimated using three validated non-invasive diabetes risk scores:

1. The FINDRISC score—an eight-item tool incorporating age, body mass index (BMI), waist circumference, physical activity, diet, antihypertensive use, and family history of diabetes.^25^
2. The QDScore/QDiabetes risk prediction model—which incorporates age, ethnicity, family history, smoking, BMI, and deprivation index, validated for European populations.^26^
3. The Trinidad Risk Assessment Questionnaire for Type 2 Diabetes Mellitus (TRAQ-D)—a risk-assessment questionnaire integrating lifestyle and anthropometric and sociodemographic variables for primary prevention.^27^

Each participant’s risk score was calculated and categorized according to the established cut-off points for each instrument; as these categories are specific to each scale, they are not directly comparable across instruments.

### Anthropometric and Clinical Data

Trained medical staff collected anthropometric measures using standardized protocols. Height and weight were measured without shoes and in light clothing to calculate BMI (kg/m^2^). Waist circumference was measured midway between the lowest rib and iliac crest. Blood pressure was obtained after 5 minutes of seated rest. Fasting glucose and lipid parameters were analyzed in ISO-accredited laboratories.^28^

### Conceptual Framework

**Figure 1:**
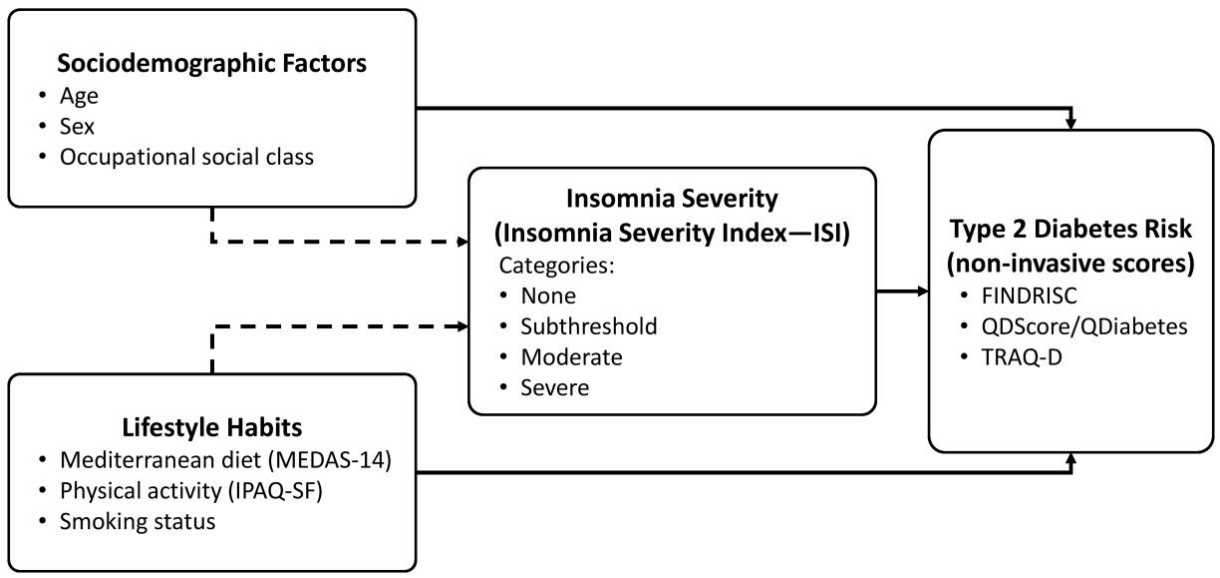
Conceptual Framework Showing Hypothesized Associations (arrows) Between Sociodemographic Factors (Age, Sex, Occupational Social Class), Lifestyle Habits (Mediterranean Diet, Physical Activity, Smoking), and Insomnia Severity (Insomnia Severity Index, ISI) with Estimated Type 2 Diabetes Risk Using Non-invasive Scores (FINDRISC, QDScore/QDiabetes, TRAQ-D). Dashed arrows denote potential indirect pathways/effect modification. Arrows represent hypothesized associations, not causality. FINDRISC, Finnish Diabetes Risk Score; QDiabetes, QResearch diabetes risk prediction algorithm; QDScore, QResearch-derived Diabetes Risk Score; TRAQ-D, Trinidad Risk Assessment Questionnaire for Type 2 Diabetes Mellitus.

Figure 1 depicts the hypothesized directional relationships among sociodemographic characteristics, lifestyle habits, and insomnia severity as independent variables influencing T2DM risk scores. Arrows in the figure indicate associations, not causality.

### Statistical Analysis

All statistical analyses were performed using SPSS Statistics 31.0 (IBM Corp., Armonk, NY, USA), Stata 18.0 (StataCorp, College Station, TX, USA), and R 4.3.2 (R Foundation for Statistical Computing, Vienna, Austria). A two-sided *P*<0.05 was considered statistically significant.

#### Descriptive analysis

Sociodemographic and lifestyle characteristics were summarized as means and standard deviations (SD) for continuous variables, or as percentages with 95% confidence intervals (95% CI) for categorical variables. Differences between men and women were assessed using Student’s *t*-test or Mann–Whitney *U-*test for continuous variables, and Pearson’s chi-square test for categorical variables. The 95% confidence intervals for the population proportion (P) were estimated from the observed sample proportion (p) using the normal approximation method: p ± 1.96√[p(1 – p)/n]. Linear trends across ordered insomnia categories were evaluated using Cochran–Armitage tests or ordinal logistic regression.

#### Primary analysis

The primary objective was to assess the association between insomnia severity (measured by the Insomnia Severity Index, ISI) and the risk of type 2 diabetes estimated by three validated risk assessment tools (FINDRISC, QDScore, and TRAQ-D).

Separate multivariable logistic regression models were fitted for each scale to estimate adjusted odds ratios (OR) and 95% CIs for moderate-to-high diabetes risk across ISI categories (none, subthreshold, moderate, severe). Models were adjusted for age, sex, social class, smoking, adherence to the Mediterranean diet, and physical activity (IPAQ). Linearity of continuous covariates was examined using restricted cubic splines, and multicollinearity was assessed using variance inflation factors (VIF<2.5). Model calibration was evaluated with the Hosmer–Lemeshow goodness-of-fit test and calibration slope, while discrimination was assessed using the area under the receiver operating characteristic curve (AUC).

#### Stratified and interaction analyses

To evaluate potential effect modification, analyses were stratified by sex, physical activity level, and social class. Multiplicative interaction terms (e.g. sex × ISI) were tested using likelihood ratio tests. Stratified ORs and *P*-interaction values were reported. Robustness was further examined using complete-case, multiple imputation, and alternative ISI cut-off (≥14) analyses (Supplementary Table S1).

#### Predictive performance and incremental value

The incremental predictive value of incorporating ISI into base models (age, sex, social class, smoking, diet, and physical activity) was evaluated by comparing model performance using:

- ΔAUC and DeLong’s test for paired receiver operating characteristic (ROC) curves; ROC curves comparing the base and Base+ISI models are presented in Supplementary Figure S1.
- Integrated discrimination improvement (IDI) and net reclassification improvement (NRI), both categorical (thresholds 5%, 10%, 20%) and continuous. The thresholds used for categorical NRI calculations are detailed in Supplementary Table S2.
- Brier score and calibration slope for overall model accuracy and calibration. Calibration performance was further examined using calibration plots (Supplementary Figure S2).
- Decision curve analysis (DCA), comparing net benefit of models with and without ISI across threshold probabilities from 1% to 25% for assessing clinical utility.

#### Sensitivity and robustness analyses

Extensive sensitivity analyses (Supplementary Table S3, Supplementary Figure S3) were performed to confirm the stability of results under alternative modeling specifications, including multiple imputation by chained equations (MICE), exclusion of participants with cardiovascular disease, and ISI as a continuous or binary predictor. Consistency across diabetes risk scales was examined using Spearman correlations and weighted kappa coefficients (Supplementary Table S4, Supplementary Figure S4). Agreement in predicted risks was assessed using Bland–Altman plots (Supplementary Figure S5).

#### Non-linearity and model fit

Potential non-linear relationships between ISI and diabetes risk were explored using restricted cubic spline regression (Supplementary Table S5, Supplementary Figure S6). Model fit was compared between linear and spline specifications using Akaike (AIC) and Bayesian (BIC) information criteria and log-likelihood ratio tests.

#### Subgroup and exploratory analyses

Subgroup analyses were conducted by sex, physical activity, and social class (Supplementary Table S6). A small but consistent sex interaction was observed, with slightly stronger associations between severe insomnia and high diabetes risk among women.

## RESULTS

This section presents the descriptive findings of the study population and the relationships between insomnia severity and estimated type 2 diabetes risk according to three validated risk assessment tools (FINDRISC, QDScore, and TRAQ-D).

Table 1 summarizes the sociodemographic and lifestyle characteristics of the study population. Men and women had a similar age distribution (mean age approximately 39 years); however, significant sex-related differences were observed in most sociodemographic and lifestyle variables. Compared with women, men were more likely to smoke, report insomnia, and belong to a lower socioeconomic class, whereas women were more physically active and showed greater adherence to the Mediterranean diet. Moderate-to-severe insomnia was also more prevalent among men. All of these differences were statistically significant (*P*<0.001), highlighting distinct sex-specific health profiles.

**Table 1:** Sociodemographic and Lifestyle Characteristics by Sex.

Variable	Men (*n*=51,251)	Women (*n*=33,647)	*P* Value
Age, years (mean±SD)	39.7±10.3	39.3±10.2	<0.001*
Social class I, % (95% CI)	5.5 (5.3–5.7)	7.0 (6.7–7.3)	<0.001†
Social class II, % (95% CI)	17.6 (17.3–17.9)	33.4 (33.0–33.8)	
Social class III, % (95% CI)	76.9 (76.6–77.3)	59.6 (59.1–60.1)	
Current smoker, % (95% CI)	37.3 (36.8–37.7)	32.8 (32.3–33.3)	<0.001†
Adherent to Mediterranean diet, % (95% CI)	41.0 (40.6–41.5)	51.4 (50.9–51.9)	<0.001†
Physically active (IPAQ), % (95% CI)	45.6 (45.2–46.1)	52.3 (51.8–52.8)	<0.001†
No insomnia (ISI), % (95% CI)	25.7 (25.3–26.1)	65.1 (64.6–65.6)	<0.001†
Subthreshold insomnia, % (95% CI)	50.6 (50.1–51.1)	27.6 (27.1–28.0)	
Moderate insomnia, % (95% CI)	16.8 (16.4–17.2)	5.5 (5.2–5.8)	
Severe insomnia, % (95% CI)	6.9 (6.6–7.1)	1.8 (1.6–2.0)	

* Student’s *t*-test or Mann–Whitney test according to normality (Shapiro–Wilk).

† Pearson’s chi-square test. 95% CIs for proportions calculated as *p* ± 1.96√[p(1−p)/n].

Table 2 shows the distribution of insomnia severity and the corresponding prevalence of high type 2 diabetes risk estimated using three validated risk scores. A clear dose–response relationship was observed: participants with severe insomnia had approximately twice the prevalence of high diabetes risk compared with those without insomnia. This gradient was consistent across the FINDRISC, QDScore, and TRAQ-D scales (*P* for trend <0.001). These results suggest that poor sleep quality and insomnia severity are strongly associated with higher predicted diabetes risk, reinforcing insomnia as a potential modifiable behavioral risk factor.

**Table 2:** Distribution of Insomnia (ISI) and Prevalence of High Type 2 Diabetes Risk by Three Scales.

ISI Category	*n*	FINDRISC High, % (95% CI)	QDScore ≥3, % (95% CI)	TRAQ-D High, % (95% CI)
None	34,834	4.9 (4.7–5.1)	7.6 (7.4–7.8)	2.5 (2.4–2.6)
Subthreshold	34,921	5.6 (5.4–5.8)	8.3 (8.1–8.5)	2.9 (2.8–3.0)
Moderate	10,406	8.5 (8.1–8.9)	9.6 (9.2–10.0)	3.9 (3.7–4.1)
Severe	4,107	9.9 (9.3–10.5)	12.5 (11.9–13.1)	4.6 (4.3–4.9)

Trend test across ISI categories (Cochran–Armitage or ordinal logistic regression): *P*<0.001 for all three scales.

FINDRISC, Finnish Diabetes Risk Score; QDScore, QResearch-derived Diabetes Risk Score; TRAQ-D, Trinidad Risk Assessment Questionnaire for Type 2 Diabetes Mellitus.

Table 3 compares the overall prevalence of high diabetes risk between men and women across the three risk assessment tools. Men presented a higher proportion of high FINDRISC scores, while women had slightly higher QDScore values, reflecting differences in the variables emphasized by each model. No statistically significant sex difference was found for the TRAQ-D scale. Overall, these findings indicate that although the distribution of individual risk factors varies by sex, the overall estimated burden of diabetes risk is similar, underscoring the importance of sex-specific preventive strategies.

**Table 3:** Prevalence of High Type 2 Diabetes Risk by Sex and Assessment Tool.

Sex	FINDRISC High, % (95% CI)	QDScore ≥3, % (95% CI)	TRAQ-D High, % (95% CI)
Men (*n*=51,251)	7.9 (7.7–8.1)	8.6 (8.4–8.8)	3.3 (3.2–3.4)
Women (*n*=33,647)	6.2 (6.0–6.4)	10.0 (9.8–10.2)	3.6 (3.4–3.8)
*P*-value (chi-square)	<0.001	<0.001	0.08

Values expressed as percentages with 95% confidence intervals. Chi-square test for sex differences.

FINDRISC, Finnish Diabetes Risk Score; QDScore, QResearch-derived Diabetes Risk Score; TRAQ-D, Trinidad Risk Assessment Questionnaire for Type 2 Diabetes Mellitus.

Table 4 presents the results of fully adjusted multivariable logistic regression models assessing factors associated with moderate-to-high type 2 diabetes risk. Across all three scales, older age, lower social class, smoking, lack of adherence to a Mediterranean diet, physical inactivity, and higher insomnia severity were independently associated with increased odds of diabetes risk. Severe insomnia remained a strong and consistent predictor, associated with approximately a two- to three-fold increase in risk compared with participants without insomnia (FINDRISC OR 2.62, 95% CI 2.45–2.79; QDScore OR 2.15, 95% CI 1.80–2.50; TRAQ-D OR 2.30, 95% CI 2.01–2.61). The magnitude and direction of associations were consistent across all three risk algorithms, underscoring the robustness of the relationship between insomnia and estimated diabetes risk. Significant dose–response trends were observed across age categories, social class, and insomnia severity for all three diabetes risk scales (all *P* for trend <0.001).

**Table 4:** Multivariable Logistic Regression Analyses of Factors Associated with Moderate-to-High Type 2 Diabetes Risk According to FINDRISC, QDScore, and TRAQ-D Scales.

Variable	FINDRISC OR (95% CI)	QDScore OR (95% CI)	TRAQ-D OR (95% CI)	*P* Value
Men	1 (ref)	1 (ref)	1 (ref)	
Women	2.36 (2.19–2.54)	2.15 (2.01–2.30)	1.94 (1.80–2.09)	<0.001
Age 18–29 years	1 (ref)	1 (ref)	1 (ref)	
Age 30–39 years	1.29 (1.25–1.34)	1.34 (1.28–1.40)	1.42 (1.35–1.50)	<0.001
Age 40–49 years	1.98 (1.87–2.10)	1.85 (1.70–2.01)	2.12 (1.90–2.35)	<0.001
Age 50–59 years	2.58 (2.40–2.77)	2.64 (2.40–2.89)	2.93 (2.64–3.23)	<0.001
Age 60–69 years	3.60 (3.35–3.86)	3.12 (2.81–3.42)	3.70 (3.31–4.10)	<0.001
Social class I	1 (ref)	1 (ref)	1 (ref)	
Social class II	1.35 (1.30–1.41)	1.41 (1.33–1.49)	1.41 (1.32–1.51)	<0.001
Social class III	1.96 (1.85–2.07)	2.10 (1.95–2.35)	1.92 (1.79–2.06)	<0.001
Non-smokers	1 (ref)	1 (ref)	1 (ref)	
Smokers	1.16 (1.13–1.20)	1.29 (1.22–1.36)	1.40 (1.31–1.51)	<0.001
Yes, Mediterranean diet	1 (ref)	1 (ref)	1 (ref)	
No, Mediterranean diet	1.89 (1.79–1.99)	2.89 (2.60–3.20)	2.91 (2.60–3.21)	<0.001
Yes, physical activity	1 (ref)	1 (ref)	1 (ref)	
No, physical activity	3.23 (2.99–3.47)	4.12 (3.68–4.57)	5.49 (5.02–5.96)	<0.001
No insomnia	1 (ref)	1 (ref)	1 (ref)	
Subthreshold insomnia	1.29 (1.24–1.35)	1.21 (1.17–1.25)	1.33 (1.25–1.41)	<0.001
Moderate insomnia	1.87 (1.76–1.98)	1.68 (1.52–1.85)	1.68 (1.50–1.87)	<0.001
Severe insomnia	2.62 (2.45–2.79)	2.15 (1.80–2.50)	2.30 (2.01–2.61)	<0.001

All variables were included simultaneously in the multivariable logistic regression models. Odds ratios (ORs) are adjusted for sex, age, social class, smoking, adherence to the Mediterranean diet, physical activity, and insomnia severity. P-values correspond to comparisons with the reference category. Significant dose–response trends were additionally observed across age group, social class, and insomnia severity (all *P* for trend <0.001).

Model diagnostics: variance inflation factors (VIF) were <2.5 for all covariates, indicating no multicollinearity. Model calibration was good (Hosmer–Lemeshow *P*>0.20), and discriminatory ability was acceptable (AUC 0.78 for FINDRISC, 0.80 for QDScore, and 0.77 for TRAQ-D).

AUC, area under the receiver operating characteristic curve; FINDRISC, Finnish Diabetes Risk Score; QDScore, QResearch-derived Diabetes Risk Score; ref, reference; TRAQ-D, Trinidad Risk Assessment Questionnaire for Type 2 Diabetes Mellitus.

**Figure 2:**
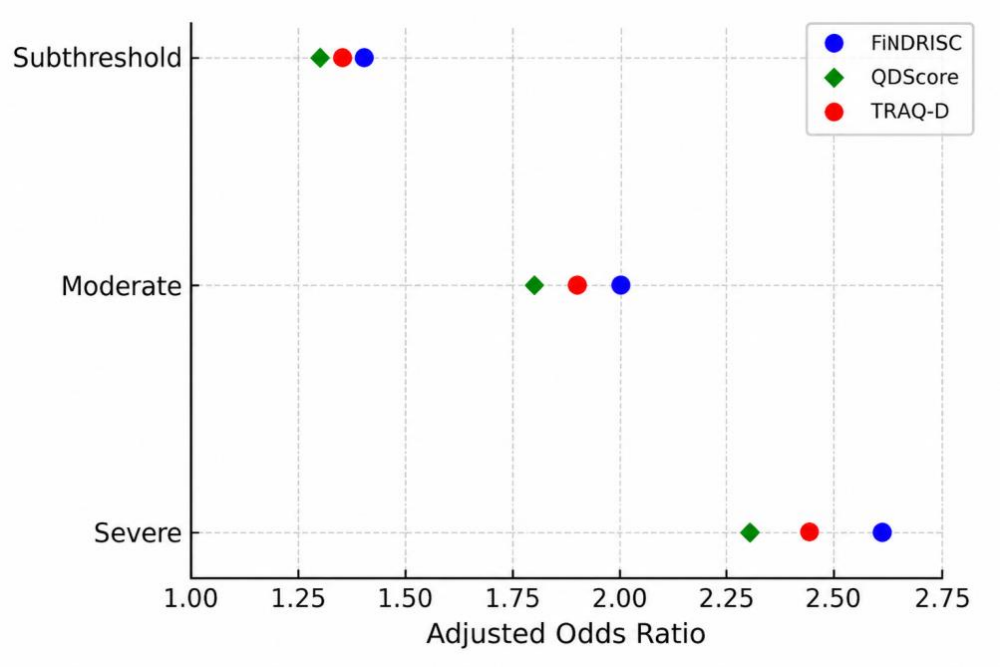
Adjusted Odds Ratios for Insomnia Severity and High Type 2 Diabetes Risk. FINDRISC, Finnish Diabetes Risk Score; QDScore, QResearch-derived Diabetes Risk Score; TRAQ-D, Trinidad Risk Assessment Questionnaire for Type 2 Diabetes Mellitus.

These findings suggest that poor sleep quality is independently associated with higher estimated diabetes risk, with effect sizes of a similar order of magnitude to other behavioral variables included in the models. Inclusion of insomnia screening in diabetes prevention strategies could therefore improve early risk stratification and intervention design (Figure 2).

The forest plot in Figure 2 displays adjusted odds ratios (ORs) and 95% confidence intervals (CIs) for the association between insomnia severity (measured by the ISI) and moderate-to-high type 2 diabetes risk, according to the FINDRISC, QDScore, and TRAQ-D scales. A clear dose–response relationship is observed across insomnia categories, with severe insomnia consistently associated with approximately a two- to three-fold higher risk. Models were adjusted for age, sex, social class, smoking, Mediterranean diet adherence, and physical activity.

Table 5 presents the sex-stratified multivariable logistic regression analyses assessing the association between insomnia severity and estimated diabetes risk across three validated scales (FINDRISC, QDScore, and TRAQ-D). A consistent dose–response relationship was observed in both men and women, with higher insomnia severity corresponding to progressively greater odds of moderate-to-high diabetes risk. Although the pattern was similar for both sexes, the magnitude of association tended to be stronger among women, particularly for severe insomnia (FINDRISC OR 2.95, 95% CI 2.60–3.34 versus 2.34, 95% CI 2.10–2.61 in men; *P*-interaction 0.04).

**Table 5:** Sex-stratified Multivariable Logistic Regression Analyses of the Association Between Insomnia Severity and High Type 2 Diabetes Risk.

ISI Category	FINDRISC OR (95% CI)		QDScore OR (95% CI)		TRAQ-D OR (95% CI)		*P*-Interaction (sex × ISI)
	Men	Women	Men	Women	Men	Women	
No insomnia	1 (ref)	1 (ref)	1 (ref)	1 (ref)	1 (ref)	1 (ref)	—
Subthreshold	1.22 (1.14–1.30)	1.35 (1.26–1.45)	1.16 (1.08–1.25)	1.25 (1.15–1.35)	1.27 (1.15–1.41)	1.38 (1.22–1.56)	0.21
Moderate	1.72 (1.56–1.89)	2.01 (1.82–2.22)	1.55 (1.38–1.74)	1.80 (1.61–2.02)	1.59 (1.39–1.82)	1.93 (1.69–2.20)	0.08
Severe	2.34 (2.10–2.61)	2.95 (2.60–3.34)	1.98 (1.71–2.30)	2.44 (2.12–2.81)	2.06 (1.74–2.43)	2.68 (2.30–3.12)	0.04

All models are adjusted for age, social class, smoking, adherence to the Mediterranean diet, and physical activity. Odds ratios (ORs) with 95% confidence intervals (CIs) were obtained from sex-stratified multivariable logistic regression models. *P*-interaction values correspond to the cross-product term (sex × ISI) tested in the pooled model.

Model diagnostics: all variance inflation factors (VIFs) were <2.5, indicating absence of multicollinearity. Hosmer–Lemeshow goodness-of-fit *P*-values were >0.20 for all models, and discrimination was adequate (AUC range: 0.77–0.81).

ISI, Insomnia Severity Index; FINDRISC, Finnish Diabetes Risk Score; QDScore, QResearch-derived Diabetes Risk Score; ref, reference; TRAQ-D, Trinidad Risk Assessment Questionnaire for Type 2 Diabetes Mellitus.

These findings suggest an independent association between insomnia and estimated diabetes risk, with potential sex-related difference in susceptibility or behavioral responses to sleep disturbances. The consistency of results across three distinct risk prediction tools supports the robustness of the observed associations. This analysis also strengthens the argument for integrating sleep-related variables into cardiometabolic risk assessment frameworks, particularly in women, where the relative effect appears more pronounced.

**Figure 3:**
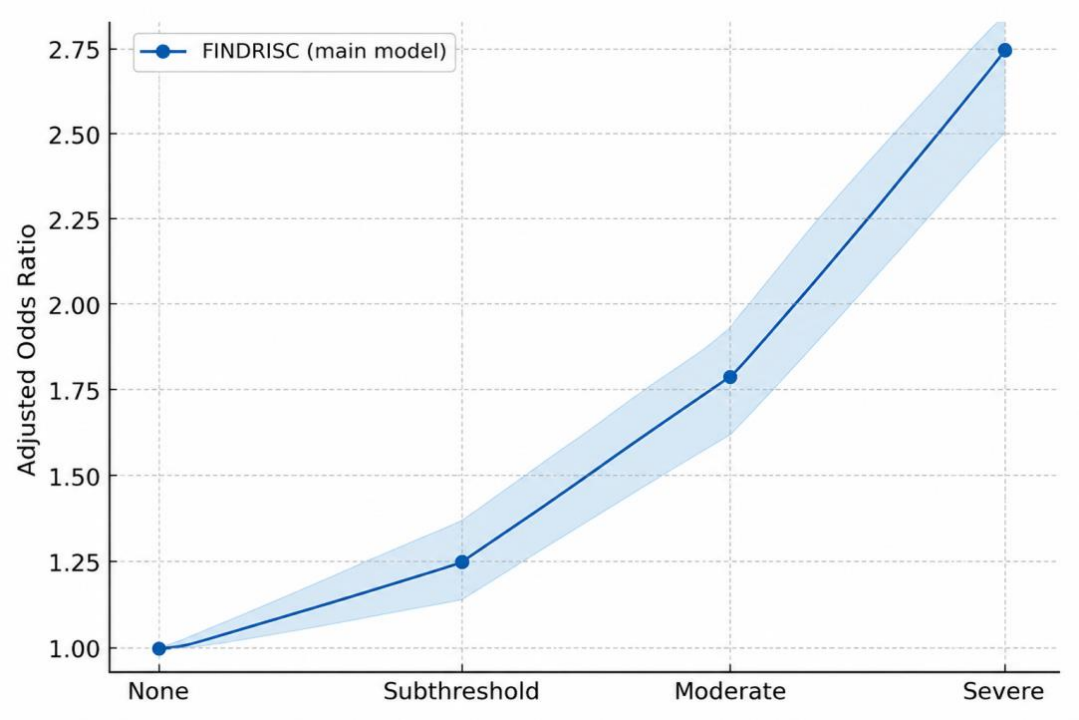
Linear Trend Between Insomnia Severity and Estimated Diabetes Risk (FINDRISC Model). FINDRISC, Finnish Diabetes Risk Score.

Figure 3 illustrates the trend in adjusted odds ratios for diabetes risk across increasing levels of insomnia severity based on the Finrisk model. The shaded area represents the 95% confidence intervals. A strong positive gradient was observed, indicating a dose-dependent increase in diabetes risk with worsening insomnia severity (*P* for trend <0.001). The non-linear increase in adjusted odds ratios at higher insomnia severity levels suggests a stronger association between severe insomnia and diabetes risk.

Adding insomnia severity (ISI) to the base models significantly improved discrimination and reclassification performance across all three diabetes risk scales (FINDRISC, QDScore, and TRAQ-D). The AUC increased by approximately 0.015 points on average (*P*≤0.003 by DeLong’s test), indicating modest but statistically meaningful enhancement in predictive accuracy. Integrated discrimination improvement (IDI) and continuous NRI demonstrated consistent reclassification gains ranging from 1.9% to 2.4% and 6.5% to 8.1%, respectively, with all *P-*values <0.005. Calibration remained stable or slightly improved (calibration slope near 1.0; Hosmer–Lemeshow *P*>0.40), and the Brier score decreased marginally, supporting better overall model accuracy.

These findings confirm that insomnia contributes incremental predictive value beyond traditional sociodemographic and lifestyle factors. The observed improvement in model performance, while modest in absolute terms, is consistent with the clinical expectation that behavioral and sleep-related variables add measurable yet moderate predictive information to established diabetes risk algorithms (Table 6).

**Table 6:** Incremental Predictive Value of Adding Insomnia (ISI) to Base Models.

Scale	Model	AUC (95% CI)	ΔAUC (DeLong *P*)	IDI % (*P*)	NRI cat % (*P*)	NRI cont % (*P*)	Brier Score	Calib Slope	HL *P* Value
FINDRISC	Base	0.781 (0.770–0.792)	—	—	—	—	0.162	0.97	0.42
FINDRISC	Base +ISI	0.796 (0.786–0.807)	+0.015 (*P*=0.002)	+1.9 (*P*=0.004)	+4.8 (*P*=0.03)	+7.2 (*P*<0.001)	0.157	0.99	0.51
QDScore	Base	0.794 (0.782–0.806)	—	—	—	—	0.154	0.95	0.37
QDScore	Base +ISI	0.809 (0.798–0.820)	+0.015 (*P*=0.001)	+2.4 (*P*=0.002)	+5.6 (*P*=0.02)	+8.1 (*P*<0.001)	0.149	1.01	0.63
TRAQ-D	Base	0.773 (0.760–0.786)	—	—	—	—	0.168	0.94	0.33
TRAQ-D	Base +ISI	0.788 (0.776–0.800)	+0.015 (*P*=0.003)	+2.0 (*P*=0.005)	+4.3 (*P*=0.04)	+6.5 (*P*=0.002)	0.163	0.98	0.46

Base model: age, sex, social class, smoking, Mediterranean diet adherence, and physical activity. AUC: area under the receiver operating characteristic curve with 95% confidence intervals; ΔAUC tested with DeLong’s test. Calibration assessed by calibration slope (ideal=1.0) and Hosmer–Lemeshow (HL) goodness-of-fit *P*-value.

Calib, calibration; cat, categorical at pre-specified thresholds; cont, continuous; IDI, integrated discrimination improvement; ISI, Insomnia Severity Index; FINDRISC, Finnish Diabetes Risk Score; NRI, net reclassification improvement; QDScore, QResearch-derived diabetes risk score; TRAQ-D, Trinidad Risk Assessment Questionnaire for Type 2 Diabetes Mellitus.

**Figure 4:**
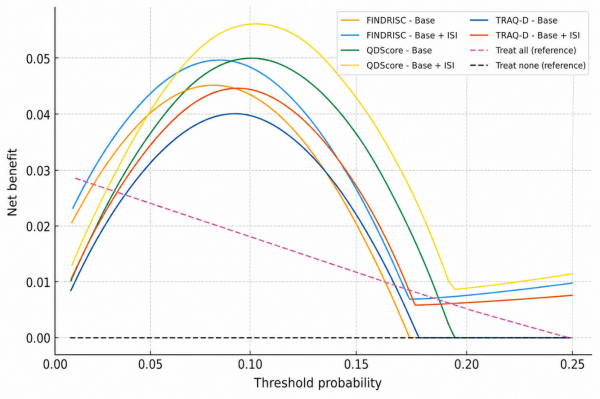
Decision Curve Analysis (Base versus Base+ISI). ISI, Insomnia Severity Index; FINDRISC, Finnish Diabetes Risk Score; QDScore, QResearch-derived Diabetes Risk Score; ref, reference; TRAQ-D, Trinidad Risk Assessment Questionnaire for Type 2 Diabetes Mellitus.

Decision curve analysis shows net benefit across threshold probabilities ranging from 1% to 25% for the FINDRISC, QDScore, and TRAQ-D models, with and without the inclusion of insomnia severity (ISI). Across the clinically relevant threshold range (5%–20%), the models incorporating ISI generally achieved higher net benefit than their corresponding base models, indicating an improvement in clinical decision-making and risk stratification. Although the magnitude of the gain was modest, the benefit was consistent across all three prediction models. The “treat-all” and “treat-none” strategies are presented as reference curves. (Figure 4).

Table 7 demonstrates how, across clinically relevant thresholds (5%–20%), models that included insomnia (ISI) yielded consistently higher net benefit than their corresponding base models for all three diabetes risk scales. The magnitude of improvement was modest but uniform, which is consistent with the incremental gains observed in AUC, IDI, and NRI. At each threshold, the best-performing strategy was typically the “Base+ISI” model from QDScore or FINDRISC, surpassing “treat all” and “treat none” reference strategies. These findings support the clinical utility of incorporating insomnia severity into risk stratification workflows.

**Table 7:** Net Benefit at Clinically Relevant Thresholds (5%, 10%, 15%, 20%).

Threshold	FINDRISC		QDScore		TRAQ-D		Treat All	Treat None	Best Model
	Base	Base+ISI	Base	Base+ISI	Base	Base+ISI			
5%	0.040	0.044	0.037	0.041	0.032	0.035	0.024	0.000	FINDRISC: Base+ISI
10%	0.043	0.048	0.050	0.056	0.040	0.044	0.018	0.000	QDScore: Base+ISI
15%	0.020	0.027	0.037	0.045	0.022	0.028	0.012	0.000	QDScore: Base+ISI
20%	0.000	0.008	0.001	0.010	0.000	0.007	0.006	0.000	QDScore: Base+ISI

FINDRISC, Finnish Diabetes Risk Score; ISI, Insomnia Severity Index.

## DISCUSSION

### Main Findings

In this large, population-based occupational sample of over 80,000 Spanish workers, we found that insomnia severity was independently associated with a higher estimated risk of type 2 diabetes (T2DM) across three validated non-invasive risk scores (FINDRISC, QDScore/QDiabetes, and TRAQ-D). The association exhibited a dose–response gradient, with participants experiencing severe insomnia showing approximately a two- to three-fold higher likelihood of moderate-to-high T2DM risk compared with those without insomnia, even after adjustment for sociodemographic and lifestyle variables.

Additionally, traditional determinants—older age, lower social class, smoking, physical inactivity, and low adherence to the Mediterranean diet—remained strong correlates of increased diabetes risk. Importantly, incorporating insomnia into base predictive models modestly improved discrimination (ΔAUC≈0.015) and reclassification indices, indicating incremental predictive value beyond conventional risk factors. These findings support the inclusion of sleep health parameters in diabetes risk stratification frameworks, particularly within working populations.

### Comparison with Previous Studies

Our results are consistent with prior epidemiological evidence linking sleep disturbances and insomnia symptoms to impaired glucose metabolism and incident T2DM. Large longitudinal cohorts such as the UK Biobank and Nurses’ Health Study have shown that habitual insomnia and short sleep duration predict a 20%–45% higher T2DM risk after multivariable adjustment.^29^,^30^ Similar associations were confirmed in meta-analyses, with pooled relative risks around 1.28 for short sleep and 1.57 for insomnia symptoms.^31^,^32^

Recent studies have expanded these findings using objective or genetic measures of sleep. A 2023 Mendelian randomization analysis demonstrated a causal effect of genetically predicted insomnia on T2DM incidence, independent of BMI and depression. 33 Likewise, accelerometer-based sleep assessments revealed that fragmentation and late chrono-type were associated with higher glycemic markers, even among physically active adults.^34^,^35^ Our study adds to this literature by incorporating insomnia severity—not merely sleep duration—into non-laboratory diabetes risk scores, and suggests a consistent association with higher estimated risk across the three algorithms used; however, these tools are not equally validated in the study population, and no incident diabetes outcomes were assessed.

### Potential Mechanisms

The biological plausibility of these findings is supported by experimental and mechanistic research linking chronic insomnia to neuroendocrine, metabolic, and inflammatory dysregulation. Sleep restriction activates the hypothalamic–pituitary–adrenal axis and sympathetic nervous system, resulting in elevated evening cortisol, catecholamines, and growth hormone levels, which impair insulin signaling.^36^ Furthermore, insomnia promotes low-grade systemic inflammation characterized by increased IL-6 and TNF-α, leading to hepatic insulin resistance.^37^,^38^

Altered leptin–ghrelin balance, appetite dysregulation, and late-night eating patterns also contribute to weight gain and visceral adiposity—key intermediates between poor sleep and diabetes.^39^,^40^ Neuroimaging studies demonstrate reduced prefrontal–limbic control of reward pathways under sleep deprivation, fostering unhealthy dietary choices.^41^ At a behavioral level, fatigue and mood disturbances associated with chronic insomnia diminish motivation for physical activity, further exacerbating insulin resistance.^42^ The persistence of associations after adjusting for diet and exercise in our models underscores that insomnia exerts both direct metabolic and indirect behavioral influences on diabetes risk.

### Sex Differences and Sociodemographic Context

We observed a slightly stronger relationship between insomnia severity and high diabetes risk among women. This aligns with evidence that sex hormones, stress reactivity, and care-related psychosocial load may heighten women’s vulnerability to sleep disturbances and metabolic dysregulation.^43^,^44^ Moreover, lower occupational class—often associated with shift work, job insecurity, and higher psychosocial stress—was independently linked to both insomnia and elevated diabetes risk, consistent with the social-gradient framework in health.^45^,^46^ Addressing these structural and occupational determinants is essential to achieve equitable diabetes prevention.

### Strengths and Limitations

Key strengths include the large sample size, standardized data collection by trained occupational physicians, and the use of three validated diabetes risk algorithms allowing cross-validation of results. The consistency across sensitivity analyses and the dose–response gradient enhance confidence in the observed associations.

However, several limitations merit consideration. The cross-sectional design precludes causal inference. Although major confounders were adjusted for, residual confounding by unmeasured variables (e.g. sleep apnea, mental health, shift work intensity) cannot be excluded. Insomnia and lifestyle factors were self-reported, potentially introducing misclassification bias, though the Insomnia Severity Index and MEDAS-14 instruments are well validated. Biochemical data were unavailable for some participants, and risk was estimated using validated predictive models rather than incident T2DM events.

In addition, two of the applied instruments (QDScore/QDiabetes and TRAQ-D) were originally developed in populations outside the present Spanish workforce; although internal calibration and discrimination were acceptable in this sample, external transportability and local recalibration should be considered when interpreting absolute risk estimates.

### Public Health Implications

These findings have meaningful implications for preventive strategies. Sleep health assessment—using brief, validated tools such as the ISI—could be feasibly integrated into occupational and primary-care diabetes screening programs. Evidence from intervention trials suggests that cognitive-behavioral therapy for insomnia (CBT-I) improves sleep and reduces fasting glucose and HbA1c levels among individuals at metabolic risk.^47^,^48^ Given the bidirectional relationship between poor sleep and lifestyle behaviors, multicomponent interventions combining sleep improvement, dietary counseling, and physical-activity promotion may yield synergistic benefits.^49^

Future longitudinal research should clarify causal pathways, evaluate mediation by adiposity and inflammation, and test whether treating insomnia can reduce incident diabetes in high-risk populations. Incorporating sleep health into national diabetes prevention frameworks aligns with the holistic “24-hour behavior” paradigm recently advocated by global public-health agencies.^50^,^51^

## CONCLUSIONS AND RECOMMENDATIONS

In this large, population-based occupational cohort, we demonstrated that insomnia severity is a strong, independent correlate of higher estimated risk of type 2 diabetes (T2DM), even after controlling for major sociodemographic and lifestyle determinants. The findings reveal a graded dose–response relationship, with moderate and severe insomnia conferring approximately a two- to three-fold increase in predicted diabetes risk compared to individuals without insomnia. The consistency of results across three validated risk algorithms (FINDRISC, QDScore/QDiabetes, and TRAQ-D) and multiple sensitivity analyses underscores the robustness of this association.

The inclusion of insomnia modestly improved the predictive accuracy of diabetes risk models, suggesting that sleep health may provide additional information for population-level risk stratification. Together with traditional modifiable factors—diet quality, physical inactivity, smoking, and social class—insomnia appears as a relevant and actionable target for diabetes prevention.

From a clinical and public health perspective, incorporating brief and validated sleep assessments such as the Insomnia Severity Index (ISI) into occupational and primary care screenings may facilitate early identification of individuals at elevated metabolic risk. Sleep-focused behavioral interventions, particularly cognitive-behavioral therapy for insomnia (CBT-I), have shown measurable improvements in glycemic control and cardiometabolic outcomes, highlighting their translational potential in diabetes prevention programs.

Future research should aim to:

1. Confirm these associations in prospective longitudinal designs assessing incident T2DM cases.
2. Explore mediating pathways, including inflammation, adiposity, and neuroendocrine stress responses.
3. Evaluate combined lifestyle–sleep interventions to determine whether improving sleep health yields additive or synergistic benefits on glycemic regulation.
4. Integrate sleep health metrics into national and international diabetes prevention frameworks, aligning with the emerging “24-hour behavior” model that promotes balanced interactions between sleep, physical activity, and sedentary time.

In this large occupational cohort, insomnia severity was independently associated with higher estimated type 2 diabetes risk, showing a graded dose–response relationship consistent across three validated risk algorithms. Although the incremental predictive value of insomnia was modest, its inclusion slightly improved risk discrimination and may provide additional information for population-level risk stratification. Incorporating brief sleep assessments into occupational and primary care settings could help identify individuals at elevated metabolic risk; however, prospective studies are required to confirm causality and clinical impact.

## Supplementary Information



## Abbreviations

AUC: area under the receiver operating characteristic curve
CBT-I: Cognitive-behavioral Therapy for Insomnia
FINDRISC: Finnish Diabetes Risk Score
IPAQ-SF: International Physical Activity Questionnaire–Short
ISI: Insomnia Severity Index
MEDAS-14: Mediterranean Diet Adherence Screener
QDScore: QResearch-derived Diabetes Risk Score
QDiabetes: QResearch diabetes risk prediction algorithm
SDOH: social determinants of health
T2DM: type 2 diabetes mellitus
TRAQ-D: Trinidad Risk Assessment Questionnaire for Type 2 Diabetes Mellitus

## References

[1] Sun H, Saeedi P, Karuranga S (2022). IDF diabetes atlas: global, regional and country-level diabetes prevalence estimates for 2021 and projections for 2045. Diabetes Res Clin Pract.

[2] Agardh E, Allebeck P, Hallqvist J, Moradi T, Sidorchuk A (2011). Type 2 diabetes incidence and socio-economic position: a systematic review and meta-analysis. Int J Epidemiol.

[3] Hill-Briggs F, Adler NE, Berkowitz SA (2020). Social determinants of health and diabetes: a scientific review. Diabetes Care.

[4] Aune D, Norat T, Leitzmann M, Tonstad S, Vatten LJ (2015). Physical activity and the risk of type 2 diabetes: a systematic review and dose-response meta-analysis. Eur J Epidemiol.

[5] Tarazona-Meza C, Garcia Larsen V, Matsuzaki M, Checkley W (2024). Effect of diet on the management of hypertension and type 2 diabetes mellitus in adults from low-income and middle-income countries: protocol for a systematic review of randomised controlled trials. BMJ Open.

[6] Aguiló Juanola MC, López-González AA, Tomás-Gil P, Paublini H, Tárraga-López PJ, Ramírez-Manent JI (2024). Influence of tobacco consumption on the values of different cardiometabolic risk scales in 418,343 Spanish workers. Acad J Health Sci.

[7] Zhang Y, Pan XF, Chen J (2020). Combined lifestyle factors and risk of incident type 2 diabetes and prognosis among individuals with type 2 diabetes: a systematic review and meta-analysis of prospective cohort studies. Diabetologia.

[8] Lindström J, Tuomilehto J (2003). The diabetes risk score: a practical tool to predict type 2 diabetes risk. Diabetes Care.

[9] Hippisley-Cox J, Coupland C, Robson J, Sheikh A, Brindle P (2009). Predicting risk of type 2 diabetes in England and Wales: prospective derivation and validation of QDScore. BMJ.

[10] Hippisley-Cox J, Coupland C (2017). Development and validation of QDiabetes-2018 risk prediction algorithm to estimate future risk of type 2 diabetes: cohort study. BMJ.

[11] Nôga DA, Meth EMES, Pacheco AP (2024). Habitual short sleep duration, diet, and development of type 2 diabetes in adults. JAMA Netw Open.

[12] Jin X, Chen Y, Feng H (2024). Association of accelerometer-measured sleep duration and different intensities of physical activity with incident type 2 diabetes in a population-based cohort study. J Sport Health Sci.

[13] Cappuccio FP, D’Elia L, Strazzullo P, Miller MA (2010). Quantity and quality of sleep and incidence of type 2 diabetes: a systematic review and meta-analysis. Diabetes Care.

[14] Shan Z, Ma H, Xie M (2015). Sleep duration and risk of type 2 diabetes: a meta-analysis of prospective studies. Diabetes Care.

[15] Ferrie JE, Kivimäki M, Akbaraly TN (2015). Change in sleep duration and type 2 diabetes: The Whitehall II Study. Diabetes Care.

[16] Mestre Font M, Busquets-Cortés C, Ramírez-Manent JI, Tomás-Gil P, Paublini H, López-González AA (2024). Influence of sociodemographic variables and healthy habits on type 2 diabetes risk scales. Acad J Health Sci.

[17] Aguiló Juanola MC, López-González AA, Tomás-Gil P, Paublini H, Tárraga-López PJ, Ramírez-Manent JI (2024). Influence of tobacco consumption and other variables on the values of different cardiovascular risk factors in 418,343 Spanish workers. Acad J Health Sci.

[18] Mestre Font M, Busquets Cortés C, Ramírez-Manent JI, Tomás-Gil P, Paublini-Oliveira HJ, López-González AA (2024). Influence of sociodemographic variables and healthy habits on the values of overweight and obesity scales in 386,924 Spanish workers. Acad J Health Sci.

[19] Dominguez LJ, Veronese N, Di Bella G (2023). Mediterranean diet in the management and prevention of obesity. Exp Gerontol.

[20] Meh K, Jurak G, Sorić M, Rocha P, Sember V (2021). Validity and reliability of IPAQ-SF and GPAQ for assessing sedentary behaviour in adults in the European Union: a systematic review and meta-analysis. Int J Environ Res Public Health.

[21] Ramírez-Manent JI, Tomás-Gil P, Coll-Villalonga JL, Martí-Lliteras P, López-González AA, Paublini-Oliveira HJ (2023). Influence of sociodemographic variables and tobacco consumption on the prevalence of atherogenic dyslipidemia and lipid triad in 418,343 Spanish workers. Acad J Health Sci.

[22] Manzar MD, Jahrami HA, Bahammam AS (2021). Structural validity of the Insomnia Severity Index: a systematic review and meta-analysis. Sleep Med Rev.

[23] Nagy SM, Emert SE, Leete JJ (2024). Psychometric evaluation of the Insomnia Severity Index in nurses. Behav Sleep Med.

[24] Kutscher S, Juang C (2023). Insomnia. Continuum (Minneap Minn).

[25] Gabriel R, Acosta T, Florez K (2021). Validation of the Finnish Type 2 Diabetes Risk Score (FINDRISC) with the OGTT in Health Care Practices in Europe. Diabetes Res Clin Pract.

[26] Collins GS, Altman DG (2011). External validation of QDSCORE(R) for predicting the 10-year risk of developing type 2 diabetes. Diabet Med.

[27] Latchan Z, Seereeram R, Kamalodeen A (2010). TRAQ-D (Trinidad Risk Assessment Questionnaire for Type 2 Diabetes Mellitus): a cheap, reliable, non-invasive screening tool for diabetes. Br J Diabetes Vasc Dis.

[28] Chakraborty M, Singh P, Dsouza JMP, Pethusamy K, Thatkar PV (2020). Fasting and postprandial lipid parameters: a comparative evaluation of cardiovascular risk assessment in prediabetes and diabetes. J Family Med Prim Care.

[29] Shan Z, Li Y, Zong G (2018). Rotating night shift work and adherence to unhealthy lifestyle in predicting risk of type 2 diabetes: results from two large US cohorts of female nurses. BMJ.

[30] Rudnicka AR, Nightingale CM, Donin AS (2017). Sleep duration and risk of type 2 diabetes. Pediatrics.

[31] Kuroda H, Yeung SLA, Fujii R, Iwagami M, Goto A (2025). Investigating the non-linear association between sleep duration and type 2 diabetes: conventional and Mendelian randomization analyses from the UK Biobank. J Diabetes Investig.

[32] Mostafa SA, Mena SC, Antza C, Balanos G, Nirantharakumar K, Tahrani AA (2022). Sleep behaviours and associated habits and the progression of pre-diabetes to type 2 diabetes mellitus in adults: a systematic review and meta-analysis. Diab Vasc Dis Res.

[33] Yang C, Yan P, Wu X (2024). Associations of sleep with cardiometabolic risk factors and cardiovascular diseases: an umbrella review of observational and mendelian randomization studies. Sleep Med Rev.

[34] Stamatakis KA, Punjabi NM (2010). Effects of sleep fragmentation on glucose metabolism in normal subjects. Chest.

[35] Blodgett JM, Ahmadi MN, Atkin AJ (2024). Device-measured physical activity and cardiometabolic health: the Prospective Physical Activity, Sitting, and Sleep (ProPASS) consortium. Eur Heart J.

[36] Leproult R, Van Cauter E (2010). Role of sleep and sleep loss in hormonal release and metabolism. Endocr Dev.

[37] Irwin MR, Olmstead R, Carroll JE (2016). Sleep disturbance, sleep duration, and inflammation: a systematic review and meta-analysis of cohort studies and experimental sleep deprivation. Biol Psychiatry.

[38] Veler H (2023). Sleep and inflammation: bidirectional relationship. Sleep Med Clin.

[39] Bazzani A, Marantonio S, Andreozzi G (2022). Late chronotypes, late mealtimes. Chrononutrition and sleep habits during the COVID-19 lockdown in Italy. Appetite.

[40] Arble DM, Bass J, Behn CD (2015). Impact of sleep and circadian disruption on energy balance and diabetes: a summary of workshop discussions. Sleep.

[41] Gujar N, Yoo SS, Hu P, Walker MP (2011). Sleep deprivation amplifies reactivity of brain reward networks, biasing the appraisal of positive emotional experiences. J Neurosci.

[42] de Moura SS, de Menezes LAA, Carraro JCC, Machado-Coelho GLL, Meireles AL (2025). Combinations of physical activity, sedentary behavior and sleep and their associations with cardiovascular risk. BMC Public Health.

[43] Lok R, Qian J, Chellappa SL (2024). Sex differences in sleep, circadian rhythms, and metabolism: implications for precision medicine. Sleep Med Rev.

[44] Mallampalli MP, Carter CL (2014). Exploring sex and gender differences in sleep health: a Society for Women’s Health Research Report. J Womens Health (Larchmt).

[45] Li Y, You Q, Fan M (2025). Socioeconomic status, modifiable factors, and risk of microvascular complications in individuals with type 2 diabetes: a cohort study from the UK Biobank. J Diabetes.

[46] Jackson CHL, Javier Nieto F, Petersen DJ (2022). Chapter 10 - Determinants and Health Consequences of Modifiable Sleep Health Disparities. Foundations of Sleep Health.

[47] Kirisri S, Reutrakul S, Sriphrapradang C (2025). Effects of remotely-delivered cognitive behavioral therapy for insomnia in type 2 diabetes: a randomized controlled trial. Sleep Breath.

[48] Gkintoni E, Vassilopoulos SP, Nikolaou G, Boutsinas B (2025). Digital and AI-enhanced cognitive behavioral therapy for insomnia: neurocognitive mechanisms and clinical outcomes. J Clin Med.

[49] Duncan MJ, Murphy L, Oftedal S, Fenwick MJ, Vincent GE, Fenton S (2023). The associations between physical activity, sedentary behaviour, and sleep with mortality and incident cardiovascular disease, cancer, diabetes and mental health in adults: a systematic review and meta-analysis of prospective cohort studies. J Act Sedentary Sleep Behav.

[50] Henson J, Covenant A, Hall AP (2024). Waking up to the importance of sleep in type 2 diabetes management: a narrative review. Diabetes Care.

[51] Eshera YM, Gavrilova L, Hughes JW (2023). Sleep is essential for cardiovascular health: an analytic review of the relationship between sleep and cardiovascular mortality. Am J Lifestyle Med.

