# Supplementary material for: Insomnia as an Independent Behavioral Correlate of Estimated Type 2 Diabetes Risk: Evidence from Three Validated Risk Assessment Scales
Source: Rambam Maimonides Med J. 2026 Jul 31;17(3):e0019. doi: 10.5041/RMMJ.10579 (PMC13436920; doi:10.5041/RMMJ.10579)
Supplement: Supplementary file 1 [file rmmj-17-3-e0019_supplement.docx]

This appendix has been provided by the authors for the benefit of readers

Supplement to Insomnia as an Independent Behavioral Predictor of Type 2 Diabetes Risk: Evidence from Three Validated Risk Assessment Scales

Ribes Valles JL, López González ÁA, Coll Campayo I, Busquets-Cortés C, Ramírez-Manent JI. Insomnia as an Independent Behavioral Predictor of Type 2 Diabetes Risk: Evidence from Three Validated Risk Assessment Scales. Rambam Maimonides Med J 2026;17(3):e0019. doi:10.5041/RMMJ.10579

# Supplementary Tables and Figures

Supplementary Table S1. Sensitivity Analyses for the Association Between Insomnia and Type 2 Diabetes Risk.

| **Model Specification** | **Definition of Insomnia** | **OR (95% CI)** | ***P*-trend** | **Comment** |
| --- | --- | --- | --- | --- |
| Main model | ISI≥15 (severe) | 2.62 (2.45–2.79) | <0.001 | Reference model |
| Alternative cut-off | ISI≥14 | 2.54 (2.39–2.70) | <0.001 | Similar results |
| ISI continuous | Per 1-point increase | 1.07 (1.05–1.09) | <0.001 | Consistent dose–response |
| Excluding CVD cases | ISI≥15 | 2.51 (2.32–2.70) | <0.001 | Robust to exclusion |
| Full imputation | ISI≥15 | 2.59 (2.41–2.77) | <0.001 | Consistent after imputation |

Sensitivity analyses using alternative definitions of insomnia and model specifications. Results are expressed as adjusted odds ratios (OR) with 95% confidence intervals (CI).

CVD, cardiovascular diseases; ISI, Insomnia Severity Index.

Supplementary Table S2.
Thresholds Used for Categorical Net Reclassification Improvement (NRI).

| **Scale** | **Low Risk (%)** | **Intermediate Risk (%)** | **High Risk (%)** |
| --- | --- | --- | --- |
| FINDRISC | 5 | 10 | 20 |
| QDScore | 5 | 10 | 20 |
| TRAQ-D | 5 | 10 | 20 |

These predefined thresholds were used to calculate categorical NRI values in Table 6. They correspond to clinically meaningful diabetes risk categories.

FINDRISC, Finnish Diabetes Risk Score; QDScore, QResearch-derived Diabetes Risk Score; TRAQ-D, Trinidad Risk Assessment Questionnaire for Type 2 Diabetes Mellitus.

Supplementary Figure S1. ROC Curves for Base and Base+ISI Models.

Receiver operating characteristic (ROC) curves comparing models with and without insomnia (ISI) across the three diabetes risk scales. Adding ISI improved area under the receiver operating characteristic curve (AUC) by approximately 0.015 for all scales, indicating better discrimination performance.

ISI, Insomnia Severity Index; FINDRISC, Finnish Diabetes Risk Score; QDScore, QResearch-derived Diabetes Risk Score; TRAQ-D, Trinidad Risk Assessment Questionnaire for Type 2 Diabetes Mellitus.

Supplementary Figure S2. Calibration Curves for Base and Base+ISI Models.

Calibration plots showing observed versus predicted diabetes risk for the Base and Base+ISI models. The inclusion of ISI did not compromise model calibration, as both versions followed the line of perfect calibration closely.

ISI, Insomnia Severity Index; FINDRISC, Finnish Diabetes Risk Score; QDScore, QResearch-derived Diabetes Risk Score; TRAQ-D, Trinidad Risk Assessment Questionnaire for Type 2 Diabetes Mellitus.

*Supplement continues on next page.*

## Robustness and Sensitivity Analyses

Supplementary Table S3. Robustness and Sensitivity Analyses.

| **Sensitivity Scenario** | **FINDRISC OR (95% CI)** | **QDScore OR (95% CI)** | **TRAQ-D  OR  (95% CI)** | **AUC FINDRISC (Base/+ISI)** | **AUC QDScore (Base/+ISI)** | **AUC TRAQ-D (Base/+ISI)** | **Notes** |
| --- | --- | --- | --- | --- | --- | --- | --- |
| Main analysis (full sample) | 2.62 (2.45–2.79) | 2.15 (1.80–2.50) | 2.30 (2.01–2.61) | 0.781/0.796 | 0.794/0.809 | 0.773/0.788 | Reference specification |
| Complete-case only | 2.60 (2.41–2.80) | 2.17 (1.83–2.56) | 2.32 (2.05–2.64) | 0.781/0.796 | 0.794/0.809 | 0.773/0.788 | No imputation; n reduced |
| Multiple imputation (MICE) | 2.59 (2.41–2.78) | 2.13 (1.79–2.49) | 2.28 (2.00–2.60) | 0.781/0.796 | 0.794/0.809 | 0.773/0.788 | 20 imputations; Rubin’s rules |
| Alternative cut-off (ISI≥14) | 2.50 (2.34–2.67) | 2.08 (1.76–2.46) | 2.24 (1.98–2.54) | 0.781/0.796 | 0.794/0.809 | 0.773/0.788 | Binary ISI at ≥14 |
| ISI continuous (per point) | — | — | — | 0.781/0.796 | 0.794/0.809 | 0.773/0.788 | Per-point effect: OR 1.07 (1.05–1.09) |
| IPAQ 3 levels (low/mod/high) | 2.64 (2.46–2.83) | 2.18 (1.84–2.59) | 2.33 (2.05–2.65) | 0.781/0.796 | 0.794/0.809 | 0.773/0.788 | Activity coded in 3 tiers |
| Mediterranean diet 0–14 score | 2.55 (2.38–2.73) | 2.12 (1.79–2.47) | 2.27 (1.99–2.59) | 0.781/0.796 | 0.794/0.809 | 0.773/0.788 | Dietary adherence as continuous score |

Adjusted odds ratios (OR) with 95% confidence intervals (CI) for severe insomnia (versus none) across alternative specifications and definitions, together with model discrimination (AUC) for Base and Base+ISI models. Models adjusted for age, sex, social class, smoking, Mediterranean diet, and physical activity unless stated.

Across all sensitivity scenarios, the association between severe insomnia and high diabetes risk remained significant, with odds ratios ranging approximately from 2.24 to 2.64 depending on the scale and specification. Model discrimination (AUC) remained stable, and the observed improvements with the inclusion of ISI were consistent with the main analysis.

AUC, area under the curve; IPAQ, International Physical Activity Questionnaire; ISI, Insomnia Severity Index; FINDRISC, Finnish Diabetes Risk Score; QDScore, QResearch-derived Diabetes Risk Score; TRAQ-D, Trinidad Risk Assessment Questionnaire for Type 2 Diabetes Mellitus.

Supplementary Figure S3. Final Forest Plot of Sensitivity Analyses.

Final refined forest plot (FINDRISC model) showing adjusted odds ratios (OR) and 95% confidence intervals for severe insomnia (versus none) across sensitivity scenarios. The *x*-axis uses decimal labels instead of scientific notation for clarity. All OR estimates remain above unity, demonstrating the robustness and stability of the association across all model specifications.

IPAQ, International Physical Activity Questionnaire; ISI, Insomnia Severity Index; MICE, Multiple Imputation by Chained Equations.

## Concordance and Subgroup Analyses

Supplementary Table S4.
Spearman Correlation Coefficients (Continuous Scores) and Weighted Kappa (High-risk Classification).

| Pair | Spearman ρ (95% CI) | Weighted κ (95% CI) | Comment |
| --- | --- | --- | --- |
| FINDRISC–QDScore | 0.76 (0.72–0.79) | 0.66 (0.60–0.71) | Moderate-to-strong agreement |
| FINDRISC–TRAQ-D | 0.68 (0.65–0.71) | 0.61 (0.60–0.71) | Moderate-to-strong agreement |
| QDScore–TRAQ-D | 0.71 (0.68–0.74) | 0.64 (0.60–0.71) | Moderate-to-strong agreement |

Correlation matrix (Spearman ρ) of the three risk scales (FINDRISC, QDScore, TRAQ-D). Values are compatible with the main analyses.

FINDRISC, Finnish Diabetes Risk Score; QDScore, QResearch-derived Diabetes Risk Score; TRAQ-D, Trinidad Risk Assessment Questionnaire for Type 2 Diabetes Mellitus.

Supplementary Figure S4. Correlation matrix (Spearman ρ) of the three risk scales (FINDRISC, QDScore, TRAQ-D).

Correlations were moderate to strong, supporting consistency across prediction tools.

FINDRISC, Finnish Diabetes Risk Score; QDScore, QResearch-derived diabetes risk score; TRAQ-D, Trinidad Risk Assessment Questionnaire for Type 2 Diabetes Mellitus.

Supplementary Figure S5a. Bland–Altman Plot: FINDRISC versus QDScore

Agreement between FINDRISC and QDScore predicted risks. Solid line: mean difference; dashed lines: ±1.96 SD limits of agreement.

FINDRISC, Finnish Diabetes Risk Score; QDScore, QResearch-derived Diabetes Risk Score.

Supplementary Figure S5b. Bland–Altman Plot: FINDRISC versus TRAQ-D

Agreement between FINDRISC and TRAQ-D predicted risks. Solid line: mean difference; dashed lines: ±1.96 SD limits of agreement.

FINDRISC, Finnish Diabetes Risk Score; QDScore, QResearch-derived Diabetes Risk Score.

Supplementary Figure S5c. Bland–Altman Plot: QDScore versus TRAQ-D.

Agreement between QDScore and TRAQ-D predicted risks. Solid line: mean difference; dashed lines: ±1.96 SD limits of agreement.

QDScore, QResearch-derived diabetes risk score; TRAQ-D, Trinidad Risk Assessment Questionnaire for Type 2 Diabetes Mellitus.

Supplementary Table S5. Linear versus Spline Model Fit.

| **Model** | AIC | BIC | **Log-likelihood** | ***P* (non-linearity)** |
| --- | --- | --- | --- | --- |
| Linear (ISI continuous) | 121,345 | 121,520 | -60,660 | — |
| Restricted cubic spline (4 knots) | 121,120 | 121,340 | -60,540 | 0.018 |

The spline-based specification provided a better fit than the linear term alone (lower AIC/BIC and improved log-likelihood), and the formal non-linearity test was statistically significant (*P*=0.018). This pattern indicates that the increase in diabetes risk with worsening insomnia is not perfectly linear: the association accelerates at higher ISI values, which is consistent with the dose–response gradients observed in categorical analyses. These results justify modeling ISI as an ordinal/continuous predictor with flexible terms in multivariable models.

AIC, Akaike information criterion; BIC, Bayesian information criterion.

Supplementary Figure S6. Adjusted Association between Insomnia Severity Index (ISI) (continuous) and High Type 2 Diabetes Risk Using a Restricted Cubic Spline.

A restricted cubic spline (4 knots) was fitted to model the adjusted log-odds of high type 2 diabetes risk as a function of the Insomnia Severity Index (ISI), controlling for age, sex, social class, smoking, Mediterranean diet adherence, and physical activity. The curve shows a monotonic increase in risk with higher ISI values, with a subtly steeper slope at moderate-to-severe scores. The shaded band denotes 95% confidence intervals. The *y*-axis is shown on a logarithmic scale to emphasize multiplicative changes in odds.

Supplementary Table S6. Effect Modification by Sex, Physical Activity, and Social Class (FINDRISC Model)

| **Interaction Term** | **Subgroup A OR (95% CI)** | **Subgroup B OR (95% CI)** | ***P* Interaction** | **Direction** | **Comment** |
| --- | --- | --- | --- | --- | --- |
| Sex × ISI (severe versus none) | Men: 2.34 (2.10–2.61) | Women: 2.95 (2.60–3.34) | 0.040 | Stronger in subgroup B | Consistent dose–response; interpret cautiously |
| IPAQ × ISI | Low/Moderate: 2.78 (2.50–3.09) | High: 2.41 (2.10–2.77) | 0.120 | Stronger in subgroup A | Consistent dose–response; interpret cautiously |
| Social class × ISI | I–II: 2.38 (2.12–2.67) | III: 2.71 (2.43–3.02) | 0.090 | Stronger in subgroup B | Consistent dose–response; interpret cautiously |

Concordance analyses showed moderate-to-strong correlations and substantial agreement between the three risk scales, supporting the consistency of the main findings. Bland–Altman plots indicated limited systematic bias and reasonable limits of agreement across pairs of instruments. Subgroup analyses suggested small-to-moderate effect modification by sex (stronger associations among women) and, to a lesser extent, by social class; no strong evidence of interaction with physical activity was found. Overall, these results reinforce the robustness and generalizability of the association between insomnia severity and estimated diabetes risk.

CI, confidence interval; IPAQ, International Physical Activity Questionnaire; ISI, Insomnia Severity Index; FINDRISC, Finnish Diabetes Risk Score.
